# A Predictive Model for Major Adverse Aortic Events in Patients with Abdominal Aortic Aneurysm Using Clinical and Biomarker Data

**DOI:** 10.3390/ijms27135946

**Published:** 2026-07-02

**Authors:** Ben Li, Farah Shaikh, Abdelrahman Zamzam, Muzammil H. Syed, Rawand Abdin, Mohammad Qadura

**Affiliations:** 1 Division of Vascular Surgery, St. Michael’s Hospital, Unity Health Toronto, University of Toronto, 30 Bond Street, Toronto, ON M5B 1W8, Canada; benx.li@mail.utoronto.ca (B.L.);; 2 Department of Surgery, University of Toronto, Toronto, ON M5S 1A1, Canada; 3 Temerty Centre for Artificial Intelligence Research and Education in Medicine (T-CAIREM), University of Toronto, Toronto, ON M5S 1A1, Canada; 4 Institute of Medical Science, University of Toronto, Toronto, ON M5S 1A1, Canada; 5 Department of Medicine, McMaster University, Hamilton, ON L8S 4L8, Canada; 6 Heart, Vascular, & Thoracic Institute, Cleveland Clinic Abu Dhabi, Abu Dhabi 112412, United Arab Emirates; 7 Li Ka Shing Knowledge Institute, St. Michael’s Hospital, Unity Health Toronto, University of Toronto, Toronto, ON M5B 1W8, Canada

**Keywords:** biomarkers, prediction, prognosis, abdominal aortic aneurysm

## Abstract

Serum biomarkers associated with abdominal aortic aneurysm (AAA) have been studied individually; however, an algorithm that considers panel of proteins to inform AAA prognosis may improve predictive accuracy. We conducted a prognostic study using a prospectively recruited cohort of patients with and without AAA (*n* = 452). Serum concentrations of seven biomarkers were measured at baseline, and the cohort was followed for 2 years. The primary outcome was major adverse aortic event (MAAE; composite of rapid AAA expansion [>0.5 cm/6 months or >1 cm/12 months] or AAA intervention). Using 10-fold cross-validation, we trained a random forest model to predict 2-year MAAE using: (1) clinical characteristics, (2) biomarkers, and (3) clinical characteristics and biomarkers. Two-year MAAE occurred in 114 (25%) patients. Four proteins were significantly elevated in patients with AAA compared to those without AAA (matrix metalloproteinase 3 [MMP-3], human epididymal secretory protein 4 [HE4/WFDC2], Chitinase 3-like-1, and Kallikrein 6/Neurosin), composing the protein panel. For predicting 2-year MAAE, our random forest model achieved an area under the receiver operating characteristic curve (AUROC) of 0.64 using clinical features alone and the addition of the four-protein panel improved performance to an AUROC of 0.80. Using a combination of clinical and biomarker data, we developed a model that accurately predicts 2-year MAAE.

## 1. Introduction

Abdominal aortic aneurysm (AAA) is a progressive cardiovascular disease defined as a dilation of the abdominal aorta to greater than 3 cm [1]. Globally, AAA results in up to 200,000 deaths annually [2]. Currently, the management of patients with AAA includes imaging surveillance to monitor aneurysm diameter [3] and surgical AAA repair is generally indicated once the diameter reaches 5.0 cm in females or 5.5 cm in males [4].

Despite clear guidelines regarding AAA screening and surveillance, current follow-up and management remain poor and many patients present with ruptured AAA requiring emergency surgery [5]. The Society for Vascular Surgery (SVS) recommends a one-time ultrasound screening for AAA in men or women aged 65–75 years with a history of tobacco use [4]. Once AAA is diagnosed, the SVS recommends the following surveillance imaging intervals based on aneurysm size: 3.0–3.9 cm (every 3 years), 4.0–4.9 cm (every 12 months), and 5.0–5.4 cm (every 6 months) [4]. However, many patients do not receive a screening ultrasound or become lost to follow up due to the intensive imaging requirements [6,7]. Furthermore, recent studies have suggested that aneurysm diameter alone is insufficient to guide the treatment of patients with AAA [8,9]. Specifically, some small AAA’s have a high risk of rupture, while some large AAA’s can remain dormant for long periods of time [10,11,12]. Importantly, there is a lack of prognostication markers for AAA. Blood-based biomarkers involved in the mechanistic pathway of aneurysm development/progression have the potential to provide additional prognostic information about aortic behavior, thereby allowing for more patient-specific management [13]. Therefore, a more practical, efficient, and accurate prognostic tool using blood-based biomarkers in combination with clinical data may improve identification of patients at high risk for adverse aortic events who would benefit from increased efforts to maintain close follow-up and subsequent intervention as needed [14].

AAA development is a dynamic process with complex pathogenesis and hallmarks include vascular smooth muscle cell apoptosis, oxidative stress, elastin fragmentation, extracellular matrix degradation, and inflammation [15]. Various biomarkers have been demonstrated to be associated with cardiovascular disease and arterial wall degeneration, including matrix metalloproteinase 3 (MMP-3) [16], human epididymal secretory protein 4 (HE4/WFDC2) [17], Chitinase 3-like-1 [18], and Kallikrein 6/Neurosin [19]. In fact, many biomarkers for cardiovascular diseases and arterial wall degeneration have been studied; however, they have not been specifically investigated as biomarkers for AAA development/progression [20,21]. The rationale for choosing the seven specific biomarkers for analysis in this study is because they have been widely investigated and have potential to be biomarkers for AAA [16,17,18,19,22].

MMP-3 is a proteolytic enzyme involved in extracellular matrix turnover and degradation of elastin and collagen within the arterial wall, processes that contribute to aneurysm formation and progression [23]. HE4/WFDC2 is a protease inhibitor associated with tissue fibrosis and collagen remodeling and has been linked to adverse cardiovascular outcomes [24]. Chitinase 3-like-1 is an inflammatory glycoprotein produced by macrophages and vascular smooth muscle cells that promotes cellular migration, angiogenesis, and vascular inflammation [25]. Kallikrein 6/Neurosin is a serine protease involved in extracellular matrix remodeling and inflammatory signaling and has been associated with cardiovascular disease severity [26]. B lymphocyte stimulator (BAFF/BLyS) is a cytokine involved in adaptive immune responses and has been linked to vascular inflammation and atherosclerosis [27]. Cathepsin S is a cysteine protease capable of degrading elastin and other extracellular matrix proteins and has been implicated in arterial wall degeneration and aneurysm development [28]. MMP-1 is a collagenase that participates in extracellular matrix breakdown and vascular remodeling [29]. Given their established involvement in biological pathways relevant to aneurysm pathogenesis, these seven biomarkers were selected as candidate predictors of AAA progression and adverse aortic outcomes [16,17,18,19,22].

Although previous studies have demonstrated correlations between these proteins and cardiovascular diseases, few have characterized their prognostic value by calculating discriminatory metrics such as area under the receiver operating characteristic curve (AUROC) [16,17,18,19,22]. Additionally, these proteins have primarily been investigated individually, with no prior exploration of the prognostic potential of a combined panel of these proteins [16,17,18,19,22]. The innovation of this project involves identifying novel biomarkers for AAA. Furthermore, given that AAA is a multifactorial and chronic disease with many metabolic pathways contributing to disease development, we propose that an integrated biomarker panel in conjunction with clinical features can achieve better accuracy in predicting AAA prognosis compared to analyzing single proteins alone [30]. By leveraging biomarker data alongside demographic and clinical characteristics associated with AAA outcomes, there is potential to develop highly accurate predictive algorithms for adverse aortic events. This study primarily focuses on combining clinical and circulating biomarker data using predictive modelling techniques with the goal of supporting the prognosis of patients with AAA to guide clinical decision-making.

## 2. Results

### 2.1. Study Population

Overall, 452 patients were included (338 with AAA and 114 without AAA). Compared to patients without AAA, those with AAA were more likely to be male (85% vs. 33%, *p* < 0.001), past or current smokers (83% vs. 60%, *p* < 0.001), and have peripheral artery disease (PAD) and/or coronary artery disease (CAD) (48% vs. 37%, *p* = 0.05). The mean aortic diameter in the AAA cohort was 4.38 (SD 0.83) cm compared to 2.29 (SD 0.44) cm in the non-AAA cohort (*p* < 0.001). The mean age was 67 (SD 8.7) years, with no difference between groups (Table 1).

### 2.2. Protein Levels

**Figure 1:**
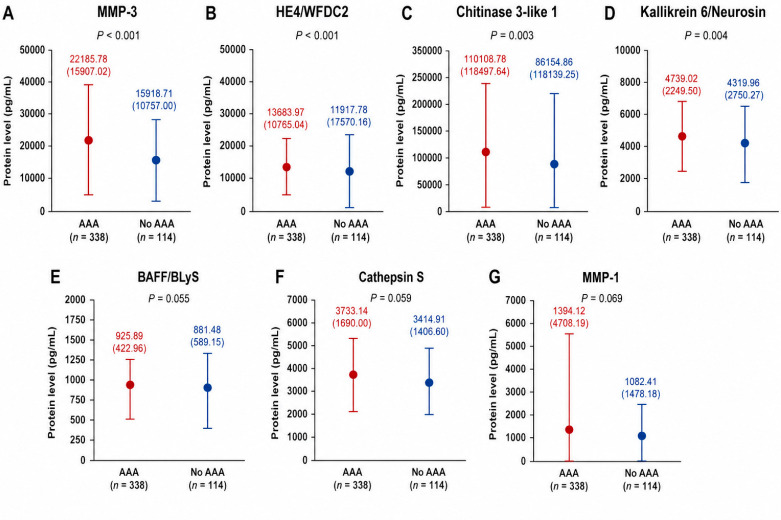
Circulating protein levels expressed as mean (standard deviation) for patients with vs. without abdominal aortic aneurysm (AAA). (**A**) Matrix metalloproteinase 3 (MMP-3), (**B**) human epididymal secretory protein 4 (HE4/WFDC2), (**C**) Chitinase 3-like-1, (**D**) Kallikrein 6/Neurosin, (**E**) B lymphocyte stimulator (BAFF/BLyS), (**F**) Cathepsin S, and (**G**) matrix metalloproteinase 1 (MMP-1).

At baseline, of the seven proteins analyzed, mean plasma levels of four proteins were significantly higher in patients with AAA compared to without AAA: MMP-3 (22,185.78 [SD 15,907.02] vs. 15,918.71 [SD 10,757.00] pg/mL, *p* < 0.001), HE4/WFDC2 (13,683.97 [SD 10,765.04] vs. 11,917.78 [SD 17,570.16] pg/mL, *p* < 0.001), Chitinase 3-like-1 (110,108.78 [SD 118,497.64] vs. 86,154.86 [SD 118,139.25] pg/mL, *p* = 0.003), and Kallikrein 6/Neurosin (4739.02 [SD 2249.50] vs. 4319.96 [SD 2750.27] pg/mL, *p* = 0.004) (Table 2, Figure 1). Since four proteins were statistically elevated in patients with AAA (MMP-3, HE4/WFDC2, Chitinase 3-like-1, and Kallikrein 6/Neurosin), this panel was further investigated.

### 2.3. Major Adverse Aortic Events over 2 Years

Complete, two-year follow-up data were available for 429 (95%) patients, with a mean duration of 22.0 (±2.1) months. There were 23 patients who were lost to follow-up despite multiple attempts to contact them. Over a 2-year follow-up period, major adverse aortic events (MAAE) occurred in 114 (34%) patients with AAA, including 32 (10%) having rapid AAA expansion and 90 (27%) requiring AAA intervention. There were no AAA ruptures observed during the follow-up period. This is most likely due to the close clinical follow-up of patients in this prospective study (Table 3).

Compared to patients who did not develop 2-year MAAE, those who had an outcome were more likely to be male (88% vs. 78%, *p* = 0.025), past smokers (59% vs. 48%, *p* = 0.003), and current smokers (30% vs. 25%, *p* = 0.002), with larger mean baseline AAA diameters (4.46 [SD 0.84] vs. 3.87 [SD 1.12] cm, *p* < 0.001) (Table 4).

### 2.4. Model Performance for Predicting MAAE

**Figure 2:**
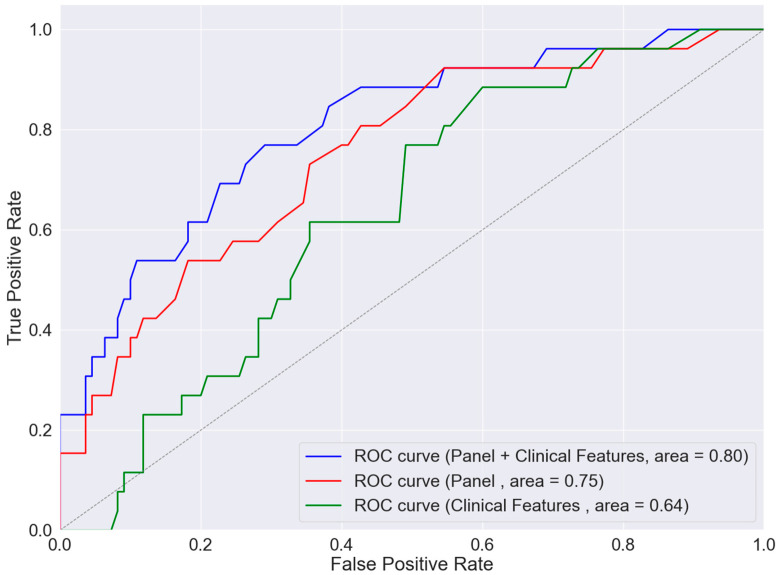
Receiver operating characteristic curve for the random forest machine learning model in predicting 2-year major adverse aortic events on test set data. Area represents area under the receiver operating characteristic curve (AUROC); panel refers to 4-protein biomarker panel consisting of matrix metalloproteinase 3 (MMP-3), human epididymal secretory protein 4 (HE4/WFDC2), Chitinase 3-like-1, and Kallikrein 6/Neurosin.

**Figure 3:**
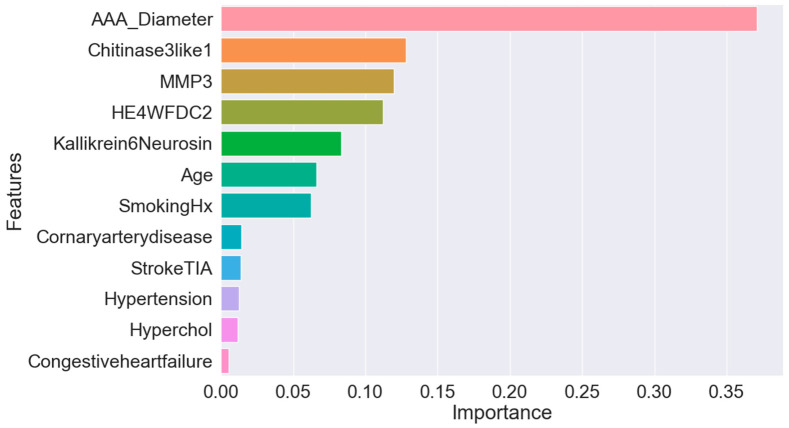
Variable importance scores (gain) for the clinical characteristics and proteins used as input features for the random forest machine learning model for predicting 2-year major adverse aortic events. Higher score indicates greater importance in contributing to an overall prediction. Abbreviations: matrix metalloproteinase 3 (MMP3), human epididymal secretory protein 4 (HE4WFDC2), AAA (abdominal aortic aneurysm), smoking history (SmokingHx; includes both past and current smoking), coronary artery disease (CAD), peripheral artery disease (PAD), hypercholesterolemia (hyperchol).

The random forest model achieved the following performance for predicting 2-year MAAE using these input features: clinical characteristics alone (area under the receiver operating characteristic curve [AUROC] 0.64), protein panel alone (AUROC 0.75), and clinical characteristics + protein panel (AUROC 0.80). The addition of the protein panel significantly improved model performance compared to clinical characteristics alone, with a net reclassification improvement (NRI) of 0.53 and integrated discrimination improvement (IDI) of 0.05 (Figure 2). The most important predictive features for 2-year MAAE were (1) baseline AAA diameter, (2) Chitinase-3 like-1, (3) MMP3, (4) HE4/WFDC2, and (5) Kallikrein 6/Neurosin (Figure 3).

### 2.5. MAAE in Low- vs. High-Risk Groups as Predicted by Model

**Figure 4:**
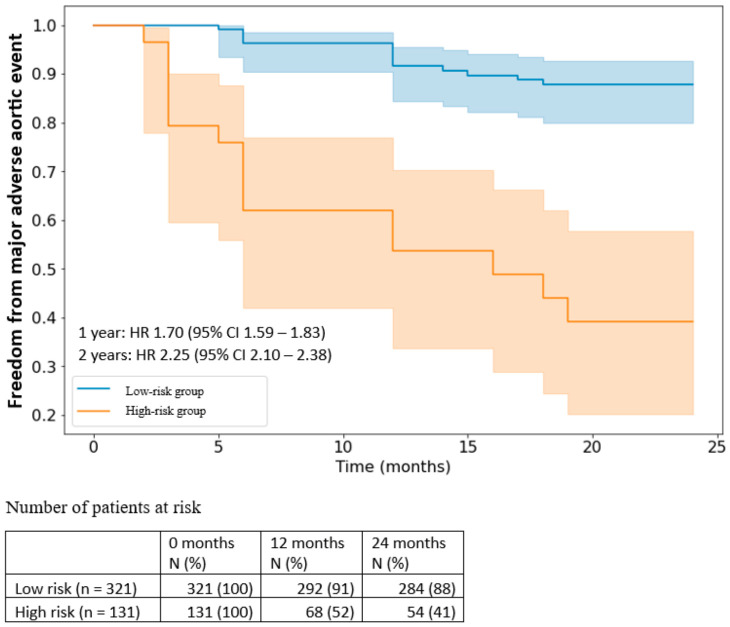
Kaplan–Meier analysis of freedom from major adverse aortic events in patients predicted to be at low vs. high risk by the random forest machine learning model. The receiver operating characteristic curve (ROC) threshold used to classify patients into low vs. high risk was 0.39. Abbreviations: HR (hazard ratio), CI (confidence interval).

The model was used to classify patients into low vs. high risk of developing MAAE based on an optimal ROC threshold of 0.39. The value of 0.39 indicates the output value on the ROC including clinical features and the four-protein panel that provides the best discrimination between patients with vs. without MAAE. On Kaplan–Meier analysis, patients predicted to be at high risk were more likely to develop MAAE at both 1-year (HR 1.70, 95% CI 1.59–1.83) and 2-years (HR 2.25, 95% CI 2.10–2.38) of follow-up (Figure 4).

### 2.6. Model Performance for Predicting MACE

**Figure 5:**
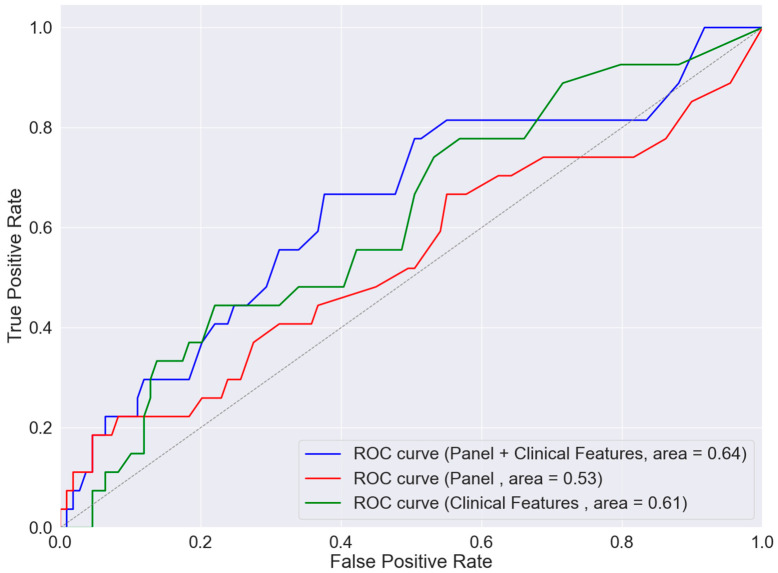
Receiver operating characteristic curve for the random forest machine learning model in predicting 2-year major adverse cardiovascular events on test set data. Area represents area under the receiver operating characteristic curve (AUROC); panel refers to 4-protein biomarker panel consisting of matrix metalloproteinase 3 (MMP-3), human epididymal secretory protein 4 (HE4/WFDC2), Chitinase 3-like-1, and Kallikrein 6/Neurosin.

In the overall cohort, 2-year major adverse cardiovascular events (MACE) occurred in 75 (17%) patients, including the following complications: myocardial infarction [MI] (*n* = 66, 16%), stroke (*n* = 8, 2%), and death (*n* = 9, 2%). There were no differences in MACE between patients with and without AAA (Table 3). The random forest model achieved the following performance for predicting 2-year MACE using these input features: clinical characteristics alone (AUROC 0.61), protein panel alone (AUROC 0.53), and clinical characteristics + protein panel (AUROC 0.64). The addition of the protein panel did not improve model performance compared to clinical characteristics alone, with an NRI of 0.10 and an IDI of 0.005 (Figure 5).

## 3. Discussion

### 3.1. Summary of Findings

In this study, we used clinical data and a targeted protein panel from a prospective cohort of 452 patients to develop models that accurately predict 2-year MAAE with an AUROC of 0.80. We showed several key findings. First, from our analysis of seven proteins, we identified four that were significantly elevated in patients with AAA compared to those without AAA. These proteins included MMP-3, HE4/WFDC2, Chitinase 3-like-1, and Kallikrein 6/Neurosin. Second, we created a panel of these four proteins and used them as input features for our random forest model. The four-protein panel performed significantly better than clinical features in predicting 2-year MAAE (AUROC 0.75 vs. 0.64). Our final model consisting of both clinical features and the four-protein panel achieved good performance with an AUROC of 0.80. The predictive power of the proteins was further demonstrated in our feature importance analysis, which demonstrated that 4 of the 5 most important predictive features were protein biomarkers (Chitinase-3 like-1, MMP3, HE4/WFDC2, and Kallikrein 6/Neurosin). Third, the four-protein panel did not have strong predictive performance for 2-year MACE (AUROC 0.53), which was lower than clinical features alone (AUROC 0.61). This suggests that the biomarkers are specific for predicting adverse aortic events, rather than cardiovascular events in general. This finding highlights that the four-protein panel is a specific marker for AAA pathology rather than a general marker to predict MACE. Fourth, we used our model to stratify patients into low vs. high risk of developing adverse aortic events. Using Kaplan–Meier analysis, we demonstrated that patients classified as high risk by our model were 2.25 times more likely to develop MAAE over a 2-year period compared to low-risk patients. This demonstrates the potential for our model to help clinicians understand the future trajectory of their patients with AAA in terms of risk of adverse aortic events.

### 3.2. Comparison to Existing Literature

Recently, Raffort and colleagues performed a systematic review summarizing predictive modelling applications in AAA [31]. The authors identified two articles that developed models to predict AAA progression, including growth rate and rupture risk [32,33]. Lee et al. (2018) analyzed 79 patients with AAA over a 2-year follow-up period and developed a support vector regression model to predict AAA growth rate using baseline aneurysm diameter and flow mediated dilatation as input features [32]. Their model predicted AAA diameter to within a 2 mm error in 71% of patients at 24 months [32]. Elsewhere, Kleinstreuer and Li (2006) developed a model using eight biomechanical parameters including maximum diameter, expansion rate, mechanical stress, diastolic pressure, asymmetry index, intraluminal thrombus, wall stiffness, and saccular index, achieving good predictive performance for rupture risk in three patients [33]. Importantly, neither study considered blood-based biomarkers as input features for their models, which have important mechanistic relationships to AAA development and progression. In our study, we used larger sample sizes (452 patients), considered more clinically important outcomes (adverse aortic events, rapid AAA expansion, and need for intervention), and included a four-protein biomarker panel specific to AAA, achieving good predictive performance with an AUROC of 0.80 for 2-year MAAE. Previously, our group demonstrated the association between HE4 and MACE in patients with AAA [34]. This study extends our prior work by validating additional circulating proteins for AAA prognosis and by assessing aortic outcomes in addition to cardiovascular events [34]. Overall, our work highlights the importance of including diverse and biologically relevant biomarkers into clinical risk prediction tools for prognosticating AAA-related outcomes.

### 3.3. Explanation of Findings

There are several potential explanations for our findings. First, our study identified 4 blood-based proteins that were important predictors of AAA prognosis. These proteins are involved in various metabolic pathways important for AAA development and progression [16,17,18,19]. MMP-3 is involved in the degradation of vascular extracellular matrix components, which is a key process in aortic wall degeneration and aneurysm development [35]. Carrell et al. (2002) demonstrated an overexpression of MMP-3 in AAA tissue, suggesting its involvement in aneurysm pathogenesis [36]. HE4/WFDC2 exhibits functional activity similar to proteinase inhibitors and is implicated in the degradation of type 1 collagen, which is important for arterial wall integrity [17]. Chitinase 3-like-1 regulates cytokine synthesis in macrophages, thereby influencing aortic smooth muscle cell migration and adhesion molecule expression in vascular endothelial cells [18]. Maegdefessel et al. (2014) showed that modulation of chitinase 3-like-1 through microRNA-24 significantly altered AAA progression in animal models [18]. Kallikrein 6 belongs to a family of secreted serine proteases with trypsin- or chymotrypsin-like activity, which has been demonstrated to be associated with cardiovascular disease outcomes in patients with diabetes [26]. Taken together, these findings explain the importance of these biomarkers in predicting AAA prognosis. Second, we found that clinical features alone were suboptimal in predicting adverse aortic events, which is corroborated by the previous literature [9,37]. This is likely because AAA progression is based on complex pathogenesis at the cellular and molecular level [38]. Therefore, it is critical to include disease-specific biomarkers when attempting to predict AAA progression and need for intervention [38]. Furthermore, we demonstrated that baseline AAA diameter was an important predictor of future adverse aortic events, which is corroborated by the previous literature [39]. Notably, baseline AAA diameter alone was not sufficient to accurately predict 2-year MAAE and the addition of circulating biomarkers significantly improved predictive performance of the model. Third, our random forest model demonstrated strong performance for several reasons. Unlike traditional statistical techniques such as logistic regression, which assume a linear correlation between independent variables and the logit of the dependent variable, advanced predictive modelling techniques are not constrained by this assumption and can effectively model complex non-linear relationships between inputs and outputs [40,41]. This is particularly important in health care data, where patient outcomes are influenced by numerous factors [42]. Due to its numerous advantages, including automation, understanding of non-linear relationships, and accurate predictions, advanced predictive modelling techniques will likely outperform traditional statistical techniques in risk prediction [40,41]. This is particularly significant in biomarker-based models, where different proteins are involved in distinct biological pathways and may interact in complex ways to contribute to a disease process [43]. In our study, random forest likely achieved good performance because it is an ensemble learning technique consisting of the aggregation of many decision trees, which (1) reduces variance, (2) handles large datasets efficiently, and (3) reduces overfitting [44]. Overall, our findings demonstrate the benefit of using a predictive model that incorporates a panel of biomarkers, leading to enhanced predictive performance compared to relying on individual biomarkers or clinical information alone. Given that AAA is a chronic and multifactorial disease involving multiple biological pathways, previous studies have highlighted the importance of a panel-based approach to improve the diagnosis and prognosis of AAA [45]. Our study confirms that by employing advanced modelling techniques to analyze clinical data alongside disease-specific biomarkers, highly accurate risk prediction tools for AAA prognosis can be developed.

### 3.4. Implications

The models developed in this study may guide clinical decision-making in several ways. In the family practice setting, once a patient screens positive for AAA on ultrasound, generalists can send a four-protein plasma panel to determine their patient’s risk for future adverse aortic events using our automated algorithm. Individuals at high risk for MAAE should be urgently referred to a vascular surgeon for further evaluation and management [46]. Those with small aneurysms at low risk for MAAE can be followed by the family physician with regular imaging based on SVS guidelines [4]. Once a referral has been made, the four-protein panel can also be used by vascular surgeons to understand a patient’s risk of developing adverse aortic events. Those at high risk may benefit from closer follow-up, more frequent imaging, and consideration of early intervention to prevent rupture [10]. Overall, our automated tool has the potential to enhance care for patients with AAA in both generalist and specialist settings. It can facilitate effective AAA risk-stratification and early identification of individuals at high risk for adverse aortic events. This, in turn, can reduce unnecessary specialist referrals and lead to improved AAA outcomes while also reducing health care costs [47]. Importantly, our tool is designed to complement, rather than replace, traditional diagnostic modalities including clinical examination and imaging by adding biological features into consideration when predicting AAA risk.

### 3.5. Limitations

There are several limitations to our study. First, this was a single center study and future validation in other institutions is needed to determine generalizability of our model. Second, although our study achieved good predictive ability considering the relatively small sample size, larger sample sizes in future studies may further improve the model’s performance. Importantly, future studies with larger cohorts are needed to reliably facilitate confidence interval estimates and calibration assessments to provide a more comprehensive evaluation of model performance and support the clinical application of risk thresholds. Third, the outcomes reported in our study were based on a 2-year follow-up period. A longer-term follow-up period may provide a better understanding of the prognostic value of our algorithm, given the chronic nature of AAA. Fourth, an additional limitation relates to the primary outcome, as no ruptures occurred during follow-up and most MAAE outcomes were attributable to the need for intervention. This likely reflects the prospective surveillance design, supporting timely elective repair. As such, the model primarily predicts clinically significant aneurysm progression and the need for intervention rather than rupture itself. Future multicenter studies with longer follow-up and more rupture events are needed to determine whether the biomarker panel independently predicts aortic rupture. Fifth, AAA progression is influenced by both molecular and hemodynamic mechanisms. Although our model incorporated clinical and biomarker data, it did not include imaging-derived features or computational fluid dynamics parameters such as aneurysm geometry and wall shear stress. Future studies integrating biological and hemodynamic markers may further improve prognostic accuracy. Finally, the biomarkers analyzed in our study are primarily used in research settings. Further translational research is needed to demonstrate the clinical value and feasibility of incorporating these biomarkers into routine care for patients with AAA.

## 4. Materials and Methods

### 4.1. Ethics Approval

This study was granted approval by the research ethics board at Unity Health Toronto, University of Toronto, Canada (REB #16-375). Written informed consent was obtained from all participants, and all procedures were conducted in accordance with the principles outlined in the Declaration of Helsinki [48]. The authors did not have access to information that could identify individual participants during or after data collection.

### 4.2. Design

This was a prognostic study using a prospectively recruited cohort and findings were reported based on the Transparent Reporting of a Multivariable Prediction Model for Individual Prognosis or Diagnosis + Artificial Intelligence (TRIPOD + AI) statement [49].

### 4.3. Patient Recruitment

This study prospectively recruited consecutive patients with and without asymptomatic infrarenal AAA who presented to vascular surgery clinics at our institution between 1 June 2018 and 1 June 2020. AAA was defined as a dilation of the abdominal aorta to ≥3 cm on any imaging modality (ultrasound, computed tomography angiography, or magnetic resonance angiography) [4]. AAA diagnosis was verified with an ultrasound performed by trained vascular laboratory technicians at our institution, which was interpreted by a board-certified vascular surgeon. The control group consisted of patients presenting with non-AAA vascular pathologies, including patients with varicose veins in addition to having an abdominal aortic diameter < 3 cm. Patients presenting with AAA diameters exceeding the operative threshold (>5 cm in females or >5.5 cm in males), symptomatic/ruptured AAA, thoracic aortic aneurysms, thoracoabdominal aortic aneurysms, aortic dissections, history of AAA repair, mycotic or inflammatory AAA were excluded.

### 4.4. Baseline Characteristics

Each patient’s clinical data, physical exam, and AAA imaging results were captured at the initial visit. Baseline clinical characteristics recorded included age, sex, hypertension (systolic blood pressure ≥ 130 mmHg, diastolic blood pressure ≥ 80 mmHg, or taking blood pressure lowering therapy [50,51]), dyslipidemia (total cholesterol > 5.2 mmol/L, triglyceride > 1.7 mmol/L, or taking lipid lowering therapy [50,51]), diabetes (hemoglobin A1c ≥ 6.5% or taking an antidiabetic medication [50,51]), current/past smoking, CAD and/or PAD, congestive heart failure (CHF), previous stroke, baseline AAA diameter, and medications (acetylsalicylic acid [ASA], statins, angiotensin-converting enzyme inhibitor (ACE-I) or angiotensin II receptor blocker (ARB), beta blocker, calcium channel blocker, hydrochlorothiazide or furosemide, oral antihyperglycemic, and insulin). Definitions for cardiovascular risk factors and medications were based on American College of Cardiology guidelines [50,51].

### 4.5. Quantification of Plasma Biomarker Levels

Blood samples were collected from patients from the median cubital vein by a trained phlebotomist in citrate and plasma was isolated with centrifugation. The plasma was aliquoted and stored at −80 °C prior to analysis with no freeze–thaw cycles. On the day of analysis, the plasma was thawed at room temperature and concentrations of 7 circulating proteins were measured in duplicate using a commercially available LUMINEX assay (Bio-Techne, Minneapolis, MN, USA) according to the manufacturer’s instructions [52]. The following proteins were chosen based on their involvement in various metabolic processes associated with cardiovascular diseases and arterial wall degeneration: MMP-3, HE4/WFDC2, Chitinase 3-like-1, Kallikrein 6/Neurosin, BAFF/BLyS, cathepsin S, and MMP-1. To minimize any inter-assay variability, all analyses were carried out on the same day. Sample intra-assay and inter-assay coefficients of variability were less than 10%, which meets the threshold for statistical acceptability [53]. Prior to sample analysis, Fluidics Verification and Calibration bead kits (Luminex Corp.) [54] were used to calibrate the MagPix analyzer (Luminex Corp.; Austin, TX, USA) [55]. At least 50 beads for each protein were acquired and analyzed using Luminex xPonent software version 4.3 [56].

### 4.6. Follow-Up and Outcomes

Outpatient clinic visits with AAA imaging surveillance were performed every 6 or 12 months following baseline assessment depending on patients’ aneurysm size based on the AAA surveillance protocol recommended by SVS AAA guidelines [4]. During these follow-up visits, changes in clinical status were noted, AAA diameter was re-measured via ultrasound, and the need for elective AAA repair was recorded. The primary outcome was 2-year major adverse aortic event (MAAE), defined as ruptured AAA, rapid AAA expansion (>0.5 cm within 6 months or >1 cm within 12 months on ultrasound from a certified vascular ultrasonographer) or need for AAA repair (open or endovascular). MAAE was chosen as the primary outcome because it includes the most clinically relevant endpoints for patients with AAA [39]. The secondary outcome was 2-year major adverse cardiovascular event (MACE), defined as myocardial infarction (MI), stroke, or death. Preliminary analysis demonstrated that all MAAE outcomes occurred in patients with AAA at baseline; therefore, prognostic models for predicting MAAE were built only on the AAA cohort. Given that MACE occurred in both AAA and non-AAA patients, prognostic models for predicting MACE were built on the whole cohort.

### 4.7. Model Development

All available demographic, clinical, and protein biomarker data were used as predictive features to build a predictive model for 2-year MAAE and MACE. The model chosen was random forest, an ensemble learning method that operates through multiple decision trees [57], which classify populations into branch-like segments to develop prediction algorithms for a target outcome using multiple covariates [58]. Given its non-parametric nature, random forest can efficiently handle large and complex datasets [58]. This model was selected because it is widely used in the literature and demonstrates good performance for predicting health outcomes [59,60,61].

**Figure 6:**
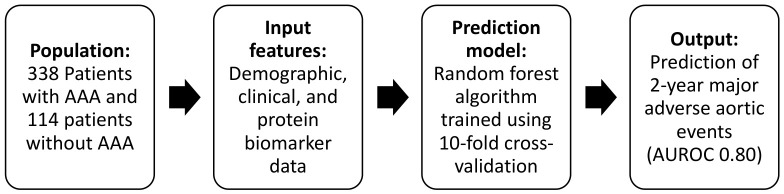
Summary of predictive model development process. AAA (abdominal aortic aneurysm), AUROC (area under the receiver operating characteristic curve).

Our cohort was randomly split into training (70%) and test (30%) sets, which is a common method for training and evaluating predictive models [62]. The random forest algorithm was trained using 10-fold cross-validation using the following input features: (1) clinical characteristics (age, sex, hypertension, dyslipidemia, diabetes, past/current smoking, CHF, CAD and/or CAD, previous stroke, baseline AAA diameter, and medications), (2) protein biomarker panel (MMP-3, HE4/WFDC2, Chitinase 3-like-1, and Kallikrein 6/Neurosin), and (3) both clinical characteristics and protein biomarker panel. The protein biomarker panel was chosen by identifying proteins that were significantly elevated in patients with AAA compared to those without AAA. Figure 6 summarizes the predictive model development process.

The reason for building and testing the models in this fashion is to understand the relative importance of the protein biomarker panel in contributing to risk predictions. This was specifically assessed using net reclassification improvement (NRI), which quantifies how well a new model correctly reclassifies subjects [63]. Specific to this study, NRI quantifies how much the addition of protein biomarkers to clinical features improves model performance for predicting AAA prognosis [63]. The integrated discrimination improvement (IDI) was also calculated for the similar purpose of assessing the added value of protein biomarkers to model performance when compared to using clinical features alone [64]. Once trained, the models were evaluated on the unseen test set and the primary model evaluation metric was area under the receiver operating characteristic curve (AUROC) [65]. The model for predicting MACE was evaluated on AAA and non-AAA patients, while the model for predicting MAAE was assessed on AAA patients only given that all MAAE outcomes occurred in the AAA cohort. The most important predictive features were determined by calculating the variable importance score (gain), a measure of the relative impact of individual covariates in contributing to an overall prediction [66].

### 4.8. Statistical Analysis

Demographic and clinical characteristics of our cohort were summarized as means and standard deviations (SDs) or numbers and proportions. Baseline differences between groups were calculated using independent *t*-test for continuous variables and chi-square test for categorical variables. Event rates for MAAE, MACE, and individual components of MAAE and MACE at 2 years were compared between AAA and non-AAA patients using a chi-square test. Protein levels were compared between patients with vs. without AAA using an independent *t*-test (if normally distributed) or Mann–Whitney U test (if non-normally distributed). Predictive ability of the model was assessed for MAAE and MACE separately. The primary metric for assessing model performance was AUROC. Using the MAAE model including both clinical features and the protein panel, patients were classified into either low or high risk of developing 2-year MAAE based on the optimal ROC threshold of 0.39. This threshold was determined by calculating the Youden Index, which is a validated summary measure of the ROC curve that provides the maximum potential effectiveness (sensitivity and specificity) of a prediction model [67]. Freedom from MAAE over 2 years in the low- vs. high-risk patients was analyzed with Kaplan–Meier curves and compared using Cox proportional hazards analysis. The purpose of this stratified analysis is to understand the potential clinical significance of the risk predictions made by the model. Specifically, it helps clinicians understand how a low- vs. high-risk patient’s trajectory over a 2-year period differs in terms of MAAE risk. Given multiple comparisons, Bonferroni correction was used to set statistical significance. All analyses were carried out using SPSS software version 23 (SPSS Inc., Chicago, IL, USA) [68].

## 5. Conclusions

In this study, we used a panel of four biomarkers in addition to clinical characteristics to develop a random forest model that accurately predicts AAA prognosis in terms of 2-year MAAE. Our algorithm accurately classified patients into low vs. high risk of adverse aortic events and can be used for AAA risk-stratification, informing clinical decisions on further vascular evaluation, specialist referrals, and surgical management, thereby supporting personalized care and improved outcomes. The four-protein panel was not significant in predicting MACE, demonstrating its specificity for predicting adverse aortic events. Furthermore, our findings shed light on avenues for future research. Clinical characteristics alone are inadequate in predicting AAA prognosis, emphasizing the significance of incorporating blood-based biomarkers into prediction models to enhance performance. This highlights the need for basic and translational studies investigating the mechanistic relationship between circulating proteins and AAA development/progression, which could strengthen our understanding of the underlying pathogenesis and potentially inform targeted treatment strategies. Importantly, our study provides impetus for conducting clinical trials that assess the impact of prediction algorithms on outcomes in patients with AAA.

## Figures and Tables

**Table 1:** Baseline clinical characteristics of patients with and without abdominal aortic aneurysm.

	Overall (*n* = 452)	No AAA (*n* = 114)	AAA (*n* = 338)	*p*
Age, years, mean (SD)	67 (8.7)	66 (10.7)	67 (7.9)	0.221
Sex, female	88 (20)	37 (33)	51 (15)	<0.001
Hypertension	312 (69)	75 (66)	237 (70)	0.387
Dyslipidemia	346 (77)	83 (73)	263 (78)	0.276
Diabetes	116 (26)	33 (29)	83 (25)	0.353
Past smoking	228 (50)	43 (38)	185 (55)	<0.001
Current smoking	120 (27)	25 (22)	95 (28)	<0.001
Congestive heart failure	18 (4)	4 (4)	14 (4)	0.765
Previous stroke	55 (13)	9 (8)	46 (14)	0.122
PAD and/or CAD	203 (45)	42 (37)	161 (48)	0.05
Baseline AAA diameter, cm, mean (SD)	4.05 (1.24)	2.29 (0.44)	4.38 (0.83)	<0.001
Acetylsalicylic acid	195 (43)	55 (48)	140 (41)	0.203
Statin	322 (71)	90 (79)	232 (69)	0.035
ACE-I/ARB	201 (45)	49 (43)	152 (45)	0.712
Beta blocker	143 (32)	36 (32)	107 (32)	0.998
Calcium channel blocker	103 (23)	23 (20)	80 (24)	0.442
Hydrochlorothiazide or furosemide	43 (10)	11 (10)	32 (10)	0.954
Oral antihyperglycemic	79 (18)	15 (13)	64 (19)	0.160
Insulin	18 (4)	2 (2)	16 (4)	0.233

Values reported as *n* (%) unless otherwise indicated. Abbreviations: PAD (peripheral artery disease), CAD (coronary artery disease), SD (standard deviation), AAA (abdominal aortic aneurysm), ACE-I (angiotensin-converting enzyme inhibitor), ARB (angiotensin II receptor blocker).

**Table 2:** Protein levels in patients with and without abdominal aortic aneurysm.

Protein	AAA (*n* = 338)		No AAA (*n* = 114)		*p*
	Mean	SD	Mean	SD	
MMP-3	22,185.78	15,907.02	15,918.71	10,757.00	<0.001
HE4/WFDC2	13,683.97	10,765.04	11,917.78	17,570.16	<0.001
Chitinase 3-like 1	110,108.78	118,497.64	86,154.86	118,139.25	0.003
Kallikrein 6/Neurosin	4739.02	2249.5	4319.96	2750.27	0.004
BAFF/BLyS	925.89	422.96	881.48	589.15	0.055
Cathepsin S	3733.14	1690	3414.91	1406.6	0.059
MMP-1	1394.12	4708.19	1082.41	1478.18	0.069

Protein levels reported in pg/mL. Abbreviations: matrix metalloproteinase (MMP), human epididymal secretory protein 4 (HE4/WFDC2), B lymphocyte stimulator (BAFF/BLyS).

**Table 3:** Event rates over 2 years.

	Overall (*n* = 452)	No AAA (*n* = 114)	AAA (*n* = 338)	*p*
Major adverse aortic event	114 (25)	0 (0)	114 (34)	<0.001
Rapid AAA expansion (>0.5 cm in 6 months or >1 cm in 12 months)	32 (7)	0 (0)	32 (10)	0.001
AAA intervention	90 (20)	0 (0)	90 (27)	<0.001
Major adverse cardiovascular event	75 (17)	60 (18)	15 (13)	0.254
Myocardial infarction	66 (15)	52 (15)	14 (12)	0.417
Stroke	8 (2)	7 (2)	1 (1)	0.403
Death	9 (2)	6 (2)	3 (3)	0.571

Values reported as *n* (%) unless otherwise indicated. Abbreviations: AAA (abdominal aortic aneurysm).

**Table 4:** Demographic and clinical characteristics of patients with and without major adverse aortic events over 2 years of follow-up.

	No MAAE (*n* = 338)	MAAE (*n* = 114)	*p*
Age, years, mean (SD)	66 (8.2)	67 (10.4)	0.091
Sex, female	74 (22)	14 (12)	0.025
Hypertension	235 (69)	77 (68)	0.692
Dyslipidemia	256 (76)	90 (79)	0.485
Diabetes	81 (24)	35 (31)	0.154
Past smoking	161 (48)	67 (59)	0.003
Current smoking	86 (25)	34 (30)	0.002
Congestive heart failure	14 (4)	4 (4)	0.765
Previous stroke	36 (11)	19 (17)	0.089
PAD and/or CAD	109 (32)	41 (36)	0.466
AAA baseline diameter, cm, mean (SD)	3.87 (1.12)	4.46 (0.84)	<0.001
Acetylsalicylic acid	155 (46)	58 (51)	0.382
Statin	253 (75)	93 (82)	0.160

Values reported as *n* (%) unless otherwise indicated. Abbreviations: PAD (peripheral artery disease), CAD (coronary artery disease), SD (standard deviation), AAA (abdominal aortic aneurysm), MAAE (major adverse aortic event).

## Data Availability

The original contributions presented in the study are included in the article; further inquiries can be directed to the corresponding author.

## References

[1] Aggarwal S., Qamar A., Sharma V., Sharma A. (2011). Abdominal Aortic Aneurysm: A Comprehensive Review. Exp. Clin. Cardiol..

[2] Wang Z., You Y., Yin Z., Bao Q., Lei S., Yu J., Xie C., Ye F., Xie X. (2022). Burden of Aortic Aneurysm and Its Attributable Risk Factors from 1990 to 2019: An Analysis of the Global Burden of Disease Study 2019. Front. Cardiovasc. Med..

[3] National Institute for Health and Care Excellence (2020). Monitoring for Abdominal Aortic Aneurysm Expansion and Risk of Rupture. Abdominal Aortic Aneurysm: Diagnosis and Management.

[4] Chaikof E.L., Dalman R.L., Eskandari M.K., Jackson B.M., Lee W.A., Mansour M.A., Mastracci T.M., Mell M., Murad M.H., Nguyen L.L. (2018). The Society for Vascular Surgery Practice Guidelines on the Care of Patients with an Abdominal Aortic Aneurysm. J. Vasc. Surg..

[5] Assar A.N., Zarins C.K. (2009). Ruptured Abdominal Aortic Aneurysm: A Surgical Emergency with Many Clinical Presentations. Postgrad. Med. J..

[6] Ahmad M., Reading K., Gannon M.X. (2021). Improving Abdominal Aortic Aneurysm (AAA) Screening Uptake through Patient Engagement-Analysis and Outcomes of Strategies to Improve Uptake at a Regional Program Level. Ann. Vasc. Surg..

[7] Chun K.C., Schmidt A.S., Bains S., Nguyen A.T., Samadzadeh K.M., Wilson M.D., Peters J.H., Lee E.S. (2016). Surveillance Outcomes of Small Abdominal Aortic Aneurysms Identified from a Large Screening Program. J. Vasc. Surg..

[8] Khan M.A., Nejim B., Faateh M., Mathlouthi A., Aurshina A., Malas M.B. (2022). Association of Abdominal Aortic Aneurysm Diameter Indexed to Patient Height with Symptomatic Presentation and Mortality. J. Vasc. Surg..

[9] Haller S.J., Azarbal A.F., Rugonyi S. (2020). Predictors of Abdominal Aortic Aneurysm Risks. Bioengineering.

[10] Nicholls S.C., Gardner J.B., Meissner M.H., Johansen H.K. (1998). Rupture in Small Abdominal Aortic Aneurysms. J. Vasc. Surg..

[11] Lancaster E.M., Gologorsky R., Hull M.M., Okuhn S., Solomon M.D., Avins A.L., Adams J.L., Chang R.W. (2022). The Natural History of Large Abdominal Aortic Aneurysms in Patients without Timely Repair. J. Vasc. Surg..

[12] Parkinson F., Ferguson S., Lewis P., Williams I.M., Twine C.P., South East Wales Vascular Network (2015). Rupture Rates of Untreated Large Abdominal Aortic Aneurysms in Patients Unfit for Elective Repair. J. Vasc. Surg..

[13] Forsythe R.O., Newby D.E., Robson J.M.J. (2016). Monitoring the Biological Activity of Abdominal Aortic Aneurysms Beyond Ultrasound. Heart.

[14] Urbonavicius S., Urbonaviciene G., Honoré B., Henneberg E.W., Vorum H., Lindholt J.S. (2008). Potential Circulating Biomarkers for Abdominal Aortic Aneurysm Expansion and Rupture—A Systematic Review. Eur. J. Vasc. Endovasc. Surg..

[15] Kuivaniemi H., Ryer E.J., Elmore J.R., Tromp G. (2015). Understanding the Pathogenesis of Abdominal Aortic Aneurysms. Expert Rev. Cardiovasc. Ther..

[16] Matusiewicz M., Rachwalik M., Krzystek-Korpacka M., Bielicki G., Berdowska I., Nowicki R., Gamian A., Jasiński M. (2020). Upregulated Sulfatase and Downregulated MMP-3 in Thoracic Aortic Aneurysm. Adv. Clin. Exp. Med..

[17] Piek A., Meijers W.C., Schroten N.F., Gansevoort R.T., de Boer R.A., Silljé H.H.W. (2017). HE4 Serum Levels Are Associated with Heart Failure Severity in Patients With Chronic Heart Failure. J. Card. Fail..

[18] Maegdefessel L., Spin J.M., Raaz U., Eken S.M., Toh R., Azuma J., Adam M., Nakagami F., Heymann H.M., Chernogubova E. (2014). miR-24 Limits Aortic Vascular Inflammation and Murine Abdominal Aneurysm Development. Nat. Commun..

[19] Chao J., Miao R.Q., Chen V., Chen L.M., Chao L. (2001). Novel Roles of Kallistatin, a Specific Tissue Kallikrein Inhibitor, in Vascular Remodeling. Biol. Chem..

[20] Sánchez-Infantes D., Nus M., Navas-Madroñal M., Fité J., Pérez B., Barros-Membrilla A.J., Soto B., Martínez-González J., Camacho M., Rodriguez C. (2021). Oxidative Stress and Inflammatory Markers in Abdominal Aortic Aneurysm. Antioxidants.

[21] Puchenkova O.A., Soldatov V.O., Belykh A.E., Bushueva O., Piavchenko G.A., Venediktov A.A., Shakhpazyan N.K., Deykin A.V., Korokin M.V., Pokrovskiy M.V. (2022). Cytokines in Abdominal Aortic Aneurysm: Master Regulators With Clinical Application. Biomark. Insights.

[22] Kato E.T., Morrow D.A., Guo J., Berg D.D., Blazing M.A., Bohula E.A., Bonaca M.P., Cannon C.P., de Lemos J.A., Giugliano R.P. (2023). Growth Differentiation Factor 15 and Cardiovascular Risk: Individual Patient Meta-Analysis. Eur. Heart J..

[23] Atkinson G., Bianco R., Di Gregoli K., Johnson J.L. (2023). The Contribution of Matrix Metalloproteinases and Their Inhibitors to the Development, Progression, and Rupture of Abdominal Aortic Aneurysms. Front. Cardiovasc. Med..

[24] Yamamoto M., Hanatani S., Araki S., Izumiya Y., Yamada T., Nakanishi N., Ishida T., Yamamura S., Kimura Y., Arima Y. (2021). HE4 Predicts Progressive Fibrosis and Cardiovascular Events in Patients With Dilated Cardiomyopathy. J. Am. Heart Assoc..

[25] Zhao T., Su Z., Li Y., Zhang X., You Q. (2020). Chitinase-3 like-Protein-1 Function and Its Role in Diseases. Signal Transduct. Target. Ther..

[26] Jaffa M.A., Bebu I., Luttrell D., Braffett B.H., Lachin J.M., Hunt K., Lopes-Virella M., Luttrell L., Lyons T.J., Jaffa A.A. (2020). Longitudinal Plasma Kallikrein Levels and Their Association With the Risk of Cardiovascular Disease Outcomes in Type 1 Diabetes in DCCT/EDIC. Diabetes.

[27] Tsiantoulas D., Sage A.P., Göderle L., Ozsvar-Kozma M., Murphy D., Porsch F., Pasterkamp G., Menche J., Schneider P., Mallat Z. (2018). B Cell-Activating Factor Neutralization Aggravates Atherosclerosis. Circulation.

[28] Wu H., Du Q., Dai Q., Ge J., Cheng X. (2018). Cysteine Protease Cathepsins in Atherosclerotic Cardiovascular Diseases. J. Atheroscler. Thromb..

[29] Mazor R., Alsaigh T., Shaked H., Altshuler A.E., Pocock E.S., Kistler E.B., Karin M., Schmid-Schönbein G.W. (2013). Matrix Metalloproteinase-1-Mediated up-Regulation of Vascular Endothelial Growth Factor-2 in Endothelial Cells. J. Biol. Chem..

[30] Pearce W.H., Shively V.P. (2006). Abdominal Aortic Aneurysm as a Complex Multifactorial Disease: Interactions of Polymorphisms of Inflammatory Genes, Features of Autoimmunity, and Current Status of MMPs. Ann. N. Y. Acad. Sci..

[31] Raffort J., Adam C., Carrier M., Ballaith A., Coscas R., Jean-Baptiste E., Hassen-Khodja R., Chakfé N., Lareyre F. (2020). Artificial Intelligence in Abdominal Aortic Aneurysm. J. Vasc. Surg..

[32] Lee R., Jarchi D., Perera R., Jones A., Cassimjee I., Handa A., Clifton D.A., Bellamkonda K., Woodgate F., Killough N. (2018). Applied Machine Learning for the Prediction of Growth of Abdominal Aortic Aneurysm in Humans. EJVES Short Rep..

[33] Kleinstreuer C., Li Z. (2006). Analysis and Computer Program for Rupture-Risk Prediction of Abdominal Aortic Aneurysms. BioMed Eng. OnLine.

[34] Khan H., Zamzam A., Shaikh F., Mamdani M., Saposnik G., Qadura M. (2025). HE4 as a Prognostic Biomarker of Major Adverse Cardiovascular Events in Patients with Abdominal Aortic Aneurysm: A Canadian Prospective Observational Study. Biomedicines.

[35] Rabkin S.W. (2017). The Role Matrix Metalloproteinases in the Production of Aortic Aneurysm. Prog. Mol. Biol. Transl. Sci..

[36] Carrell T.W.G., Burnand K.G., Wells G.M.A., Clements J.M., Smith A. (2002). Stromelysin-1 (Matrix Metalloproteinase-3) and Tissue Inhibitor of Metalloproteinase-3 Are Overexpressed in the Wall of Abdominal Aortic Aneurysms. Circulation.

[37] Spinelli D., Benedetto F., Donato R., Piffaretti G., Marrocco-Trischitta M.M., Patel H.J., Eagle K.A., Trimarchi S. (2018). Current Evidence in Predictors of Aortic Growth and Events in Acute Type B Aortic Dissection. J. Vasc. Surg..

[38] Golledge J. (2019). Abdominal Aortic Aneurysm: Update on Pathogenesis and Medical Treatments. Nat. Rev. Cardiol..

[39] Ahmed R., Ghoorah K., Kunadian V. (2016). Abdominal Aortic Aneurysms and Risk Factors for Adverse Events. Cardiol. Rev..

[40] Stoltzfus J.C. (2011). Logistic Regression: A Brief Primer. Acad. Emerg. Med..

[41] Kia B., Mendes A., Parnami A., George R., Mobley K., Ditto W.L. (2020). Nonlinear Dynamics Based Machine Learning: Utilizing Dynamics-Based Flexibility of Nonlinear Circuits to Implement Different Functions. PLoS ONE.

[42] Chatterjee P., Cymberknop L.J., Armentano R.L. (2019). Nonlinear Systems in Healthcare towards Intelligent Disease Prediction. Nonlinear Systems—Theoretical Aspects and Recent Applications.

[43] Smith B.J., Silva-Costa L.C., Martins-de-Souza D. (2021). Human Disease Biomarker Panels through Systems Biology. Biophys. Rev..

[44] Couronné R., Probst P., Boulesteix A.-L. (2018). Random Forest versus Logistic Regression: A Large-Scale Benchmark Experiment. BMC Bioinform..

[45] Molacek J., Treska V., Zeithaml J., Hollan I., Topolcan O., Pecen L., Slouka D., Karlikova M., Kucera R. (2019). Blood Biomarker Panel Recommended for Personalized Prediction, Prognosis, and Prevention of Complications Associated with Abdominal Aortic Aneurysm. EPMA J..

[46] Robinson D., Mees B., Verhagen H., Chuen J. (2013). Aortic Aneurysms—Screening, Surveillance and Referral. Aust. Fam. Physician.

[47] Margolis J., Barron J.J., Grochulski W.D. (2005). Health Care Resources and Costs for Treating Peripheral Artery Disease in a Managed Care Population: Results from Analysis of Administrative Claims Data. J. Manag. Care Pharm..

[48] World Medical Association (2013). World Medical Association Declaration of Helsinki: Ethical Principles for Medical Research Involving Human Subjects. JAMA.

[49] Collins G.S., Moons K.G.M., Dhiman P., Riley R.D., Beam A.L., Calster B.V., Ghassemi M., Liu X., Reitsma J.B., van Smeden M. (2024). TRIPOD+AI Statement: Updated Guidance for Reporting Clinical Prediction Models That Use Regression or Machine Learning Methods. BMJ.

[50] Grundy S.M., Stone N.J., Bailey A.L., Beam C., Birtcher K.K., Blumenthal R.S., Braun L.T., de Ferranti S., Faiella-Tommasino J., Forman D.E. (2019). 2018 AHA/ACC/AACVPR/AAPA/ABC/ACPM/ADA/AGS/APhA/ASPC/NLA/PCNA Guideline on the Management of Blood Cholesterol. J. Am. Coll. Cardiol..

[51] Whelton P.K., Carey R.M., Aronow W.S., Casey D.E., Collins K.J., Dennison H.C., DePalma S.M., Gidding S., Jamerson K.A., Jones D.W. (2018). 2017 ACC/AHA/AAPA/ABC/ACPM/AGS/APhA/ASH/ASPC/NMA/PCNA Guideline for the Prevention, Detection, Evaluation, and Management of High Blood Pressure in Adults. J. Am. Coll. Cardiol..

[52] Luminex Assays, Multiplex Immunoassays. https://www.bio-techne.com/.

[53] Reed G.F., Lynn F., Meade B.D. (2002). Use of Coefficient of Variation in Assessing Variability of Quantitative Assays. Clin. Diagn. Lab. Immunol..

[54] Luminex Assays—CA. https://www.thermofisher.com/ca/en/home/life-science/antibodies/immunoassays/procartaplex-assays-luminex.html.

[55] MAGPIX^®^ System|xMAP Instrument|Luminex Corporation. https://www.luminexcorp.com/magpix-system/.

[56] Luminex Corporation (2021). xPONENT^®^ Software for xMAP^®^ Instruments.

[57] Rigatti S.J. (2017). Random Forest. J. Insur. Med..

[58] Song Y.-Y., Lu Y. (2015). Decision Tree Methods: Applications for Classification and Prediction. Shanghai Arch. Psychiatry.

[59] Elfanagely O., Toyoda Y., Othman S., Mellia J.A., Basta M., Liu T., Kording K., Ungar L., Fischer J.P. (2021). Machine Learning and Surgical Outcomes Prediction: A Systematic Review. J. Surg. Res..

[60] Bektaş M., Tuynman J.B., Costa Pereira J., Burchell G.L., van der Peet D.L. (2022). Machine Learning Algorithms for Predicting Surgical Outcomes after Colorectal Surgery: A Systematic Review. World J. Surg..

[61] Senders J.T., Staples P.C., Karhade A.V., Zaki M.M., Gormley W.B., Broekman M.L.D., Smith T.R., Arnaout O. (2018). Machine Learning and Neurosurgical Outcome Prediction: A Systematic Review. World Neurosurg..

[62] Xu Y., Goodacre R. (2018). On Splitting Training and Validation Set: A Comparative Study of Cross-Validation, Bootstrap and Systematic Sampling for Estimating the Generalization Performance of Supervised Learning. J. Anal. Test..

[63] Kerr K.F., Wang Z., Janes H., McClelland R.L., Psaty B.M., Pepe M.S. (2014). Net Reclassification Indices for Evaluating Risk-Prediction Instruments: A Critical Review. Epidemiology.

[64] Pencina M.J., Demler O.V. (2012). Novel Metrics for Evaluating Improvement in Discrimination: Net Reclassification and Integrated Discrimination Improvement for Normal Variables and Nested Models. Stat. Med..

[65] Hajian-Tilaki K. (2013). Receiver Operating Characteristic (ROC) Curve Analysis for Medical Diagnostic Test Evaluation. Casp. J. Intern. Med..

[66] Loh W.-Y., Zhou P. (2021). Variable Importance Scores. J. Data Sci..

[67] Ruopp M.D., Perkins N.J., Whitcomb B.W., Schisterman E.F. (2008). Youden Index and Optimal Cut-Point Estimated from Observations Affected by a Lower Limit of Detection. Biom. J..

[68] SPSS Software. https://www.ibm.com/analytics/spss-statistics-software.

